# Superantigens and Streptococcal Toxic Shock Syndrome

**DOI:** 10.3201/eid0910.030042

**Published:** 2003-10

**Authors:** Thomas Proft, Shiranee Sriskandan, Lily Yang, John D. Fraser

**Affiliations:** * University of Auckland, Auckland, New Zealand; † Imperial College, London, United Kingdom

## Abstract

Superantigens produced by *Streptococcus pyogenes* have been implicated with streptococcal toxic shock syndrome (STSS). We analyzed 19 acute-phase serum samples for mitogenic activity from patients with severe streptococcal disease. The serum samples from two patients in the acute phase of STSS showed strong proliferative activity. Streptococcal mitogenic exotoxin (SME) Z-1 and streptococcal pyrogenic exotoxin (SPE)-J were identified in one patient with peritonitis who recovered after 2 weeks in intensive care. SMEZ-16 was found in a second patient who died on the day of admission. Sequential serum samples taken on day 3 after admission from patient 1 showed clearance of mitogenic activity but absence of neutralizing anti-SMEZ antibodies. Serum samples taken on day 9 from this patient showed evidence of seroconversion with high levels of anti-SMEZ antibodies that neutralized SMEZ-1 and 12 other SMEZ-variants. These results imply that a high level of SMEZ production by group A streptococcus is a causative event in the onset and subsequent severity of STSS.

Since the 1980s, a marked increase has occurred in highly invasive group A streptococcal (GAS) infections, in particular streptococcal toxic shock syndrome (STSS) associated with necrotizing fasciitis or myositis (*1**–**4*). The classical case definition for STSS is similar to staphylococcal toxic shock, caused by *Staphylococcus aureus,* but the outcome is more serious in STSS, with a reported death rate of 30% to 70% (*2**,**5**,**6*).

The multiorgan involvement in STSS suggests that a toxin produced by GAS might be involved in pathogenesis. Prime candidates are the streptococcal superantigens (SAgs), a family of highly mitogenic proteins secreted individually or in certain combinations by many *Streptococcus pyogenes* strains (*7**–**10*), although other virulence factors, such as Streptolysin O and various cell wall antigens can also cause toxic shock (*11*). Superantigens simultaneously bind to major histocompatibility complex class II molecules and T-cell receptor molecules bearing a particular V-β region. This binding results in the activation of a large proportion of antigen-presenting cells and T cells, with subsequent release of high systemic levels of cytokines (*12**–**15*).

Several lines of evidence support the hypothesis of SAg involvement in STSS. Toxic shock syndrome (TSS) toxin, produced by *S. aureus*, has been associated with most menstrual TSS cases (*7*). TSS toxin is a typical SAg that is functionally and structurally related to the staphylococcal and streptococcal SAgs (*16*). Moreover, animal models have shown that TSS toxin and other SAgs induce TSS-like symptoms in rabbits and rodents (*17**,**18*). The lack of neutralizing anti-SAg antibodies appears to be a key risk factor for the development of staphylococcal and streptococcal toxic shock (*19**,**20*).

The major cytokines released from antigen-presenting cells and T cells after activation by SAgs are tumor necrosis factor alpha (TNF-α), tumor necrosis factor beta (TNF-β), interleukin (IL)-1, and IL-2 (*11**–**14*). TNF-α is the prime mediator of shock; anti–TNF-α has been shown to inhibit the progression of SAg-driven shock in mice and baboons (*17**,**18**,**21*).

In contrast to TSS toxin and staphylococcal TSS, the association of individual streptococcal SAgs to STSS is much less understood. Several studies described the potential involvement of streptococcal pyrogenic exotoxin (SPE) A in invasive streptococcal disease (*2**,**19**,**22**,**23*), while others reported an association with SPE-C (*24**,**25*). In addition, some cases of STSS are not associated with SPE-A or SPE-C (*26*). Notably, these studies were performed without knowledge of other streptococcal SAgs that are now known to exist.

Superantigen activity found in acute-phase serum samples from streptococcal disease patients has been reported. (In this article, the term “acute-phase serum” refers to serum taken on the day of admission). Sriskandan et al. published a study of seven patients with severe streptococcal infections: SPE-A was detected in serum samples from four patients (*27*). Recently, Norby-Teglund and Berdal reported a strong proliferative response in an acute-phase serum sample collected from a patient with STSS, indicating that the sample contained an unknown SAg (*28*). Mitogenic activity was also detected in serum samples from mice infected with a SAg-producing *S. pyogenes* strain (*29*).

Since these reports, several novel streptococcal SAgs have been identified, including streptococcal mitogenic exotoxin Z (SMEZ; *30*), SMEZ-2, SPE-G, SPE-H (*31*), SPE-J, and SPE-I (*32*), all possessing typical SAg features and highly mitogenic on human T cells. In addition, several variants of SMEZ showed significant antigenic variation (*33*). These findings suggest that, in addition to SPE-A and SPE-C, one or more of these novel toxins might be involved in STSS.

All known streptococcal SAgs (with the exception of SMEZ, SPE-G, and SPE-J) are localized on mobile DNA elements (*34*). As a consequence, each GAS isolate usually carries the genes for SMEZ, SPE-G, and SPE-J, plus a certain combination of other *sag* genes. Not much is known about the control of *sag* gene expression, but a recent study indicates that an unknown host factor is involved in the control of SPE-C expression (*35*)

We analyzed serum samples from 19 patients with severe streptococcal infections for mitogenic activity to identify bioactive SAgs and find a correlation between SAg activity and disease severity. In addition, we genotyped the matching streptococcal isolates from these patients for all known streptococcal *sag* genes and tested them for their ability to produce SAg protein in vitro.

## Material and Methods

### Patient Serum Samples and Streptococcal Isolates

We included serum samples from all 21 patients referred to the Hammersmith Hospital’s Infectious Diseases service from November 1994 to November 2000 who had microbiologically confirmed invasive GAS disease and required hospital admission. Two patients who used intravenous drugs were subsequently excluded to reduce the risk for bloodborne viruses. Aliquots of serum (residual to serum required for clinical purposes) were separated from blood drawn for clinical purposes and frozen immediately at –70°C before testing for mitogens or antibodies. Samples were obtained at the point of admission to hospital (at initiation of antibiotic therapy) and then on sequential days during treatment up to a maximum of 10 days. Streptococcal isolates were cultured directly from blood or tissue, identified by the hospital diagnostic laboratory, and then cultured once in Todd Hewitt broth before immediate freezing in 15% glycerol and before growth for SAg analysis. All 19 patients had invasive streptococcal disease; patients with STSS were identified by using standard criteria (*1**–**3*) (Table). The study was approved by the Hammersmith Hospital Research Ethics Committee.

**Table:** Summary of the results from *sag* genotyping and SAg in vitro expression^a,b^

Serum	Isolate	Focus	*spe-a*	SPE-A	*sag* gene/SAg protein production															SMEZ^c^
					*spe-c*		SPE-C	*spe-g*	SPE-G	*spe-h*	SPE-H	*spe-i*	SPE-I	*spe-j*	SPE-J	*smez*	SMEZ	*ssa*	SSA	
Focal infection/bacteremia with STSS																				
94/31	H292	Fasciitis	-	-	-		-	+	-	+	-	+	++	+	-	+	-	-	-	+
95/02	H293	Fasciitis	-	-	-		-	+	-	+	-	-	-	+	-	+	-	-	-	++
96/2	H297	Peritonitis	+	-	-		-	+	++	-	-	-	-	+	+	+	-	-	-	+
98/5	H325	Fasciitis	+	+++	-		-	+	-	-	-	-	-	+	-	+	-	+	-	+
98/8	H327	Cellulitis	-	-	-		-	+	+	-	-	-	-	+	-	+	_	+	+	-
98/11	H330	Endometritis	+	+++	-		-	+	-	-	-	-	-	+	+	+	-	+	-	+
99/1	H360	Occult bact.	-	-	+		+	+	+	-	-	-	-	+	-	+	-	-	-	++
99/18	H366	Pneumonia	+	+++	-		-	+	-	-	-	-	-	+	++	+	-	-	-	+
20/07	H378	Pneumonia	-	-	-		-	+	-	+	-	-	-	+	-	+	-	+	-	+
Focal infection/bacteremia, no STSS																				
95/8	H295	Cellulitis	-	-		-	-	+	-	-	-	-	-	+	+++	+	-	-	-	++
98/1	H319	Cellulitis	-	-		-	-	+	++	-	-	-	-	+	++	+	-	-	-	++
20/01	H369	Pelvic clot	-	-		-	-	+	-	-	-	-	-	+	+	+	-	-	-	+++
20/04	H370	Thromobosis	-	-		-	-	+	+	-	-	-	-	+	-	+	-	-	-	-
Focal infection, no bacteremia, no STSS																				
97/15	H307	Bursitis	-	-		+	+++	+	-	-	-	-	-	+	+	+	-	-	-	+
97/18	H308	Cellulitis	+	-		-	-	+	-	+	-	-	-	+	-	+	-	+	-	-
97/21	H311	Cellulitis	-	-		-	-	+	-	-	-	-	-	+	-	+	-	-	-	+
97/23	H314	Cellulitis	+	-		-	-	+	+	+	-	+	-	+	+	+	-	-	-	++
97/24	H315	Cellulitis	+	-		-	-	+	-	-	-	-	-	+	+	+	-	-	-	-
97/26	H316	Amnionitis	-	-		+	+++	+	+	-	-	-	-	+	++	+	-	+	-	-

^a^SAg, superantigen; SPE, streptococcal pyrogenic exotoxin; SME, streptococcal mitogenic exotoxin; STSS, streptococcal toxic shock syndrome. ^b^*S. pyogenes* isolates from patients with and without STSS were genotyped by polymerase chain reaction with *sag* specific primers. Concentrated supernatant from the in vitro cultured isolates were analyzed for secreted SAgs by using Western blot analysis with recombinant SAg standards. SMEZ expression was also analyzed by using the more sensitive Jurkat cell assay, which has a threshold of approximately 10 pg/mL. SAg expression by Western blot: -, no detectable protein; +, <2 ng/mL; ++, 2–10 ng/mL; +++, >10 ng/mL. SMEZ expression by Jurkat assay: -, no detectable SMEZ; +, >10 pg/ml (10,000–20,000 cpm); ++, 20,000–30,000 cpm; +++, >30,000 cpm. ^c^Jurkat assay.

### Toxin Proliferation Assay

Human peripheral blood lymphocytes (PBLs) were purified from blood of a healthy donor by using Histopaque Ficoll (Sigma Chemical Co., St. Louis, MO) fractionation. PBLs were incubated in 96-well, round-bottom microtiter plates at 10^5^ cells per well with RPMI-10 (RPMI with 10% fetal calf serum [FCS]) containing varying dilutions of recombinant toxins. After 3 days, 0.1 μCi [^3^H] thymidine was added to each well and cells were incubated for another 24 h. Cells were harvested and counted on a scintillation counter.

Jurkat cells (a human T-cell line) and LG-2 cells (a human B-lymphoblastoid cell line, homozygous for HLA-DR1) were harvested in log phase and resuspended in RPMI-10. One hundred microliters of the cell suspension, containing 1x10^5^ Jurkat cells and 2x10^4^ LG-2 cells was mixed with 100 μL of *S. pyogenes* culture supernatant (undiluted, 1:10, 1:100) on 96-well plates. After incubating overnight at 37°C, 100-μL aliquots were transferred onto a fresh plate and 100 μL (1x10^4^) of SeI cells (IL-2 dependent murine T-cell line) per well was added. After incubating for 24 h, 0.1 μCi [^3^H] thymidine was added to each well, and cells were incubated for another 24 h. Cells were harvested and counted on a scintillation counter. As a control, a dilution series of IL-2 was incubated with SeI cells.

PBLs were obtained and stimulated as described under toxin proliferation assay above, with the exception that the 10% FCS was replaced by 5% FCS plus 5% patient serum. All recombinant toxins were used at subsaturating concentrations, which were 0.05 ng/mL (SMEZ-2), 0.1 ng/mL (all other SMEZ variants, SPE-C, SPE-I, SPE-J, streptococcal superantigen [SSA]), 1 ng/mL (SPE-G), 2 ng/mL (SPE-A), and 10 ng/mL (SPE-H).

PBLs from a single donor were used for all tests. We determined the neutralizing response by comparing the T-cell proliferation with a control test using 10% FCS instead of 5% patient serum plus 5% FCS. The relative inhibition was calculated as 1 cpm (patient serum) per cpm (FCS).

*S. pyogenes* isolates were grown overnight in 10 mL of brain heart infusion (BHI) medium (Difco Laboratories, Detroit, MI) at 37°C in 15-mL Falcon tubes without agitation. The cells were spun down and washed, and the genomic DNA was extracted as described previously (*33*). The purified DNA was resuspended in 50 μL of Tris-EDTA buffer and used for polymerase chain reaction (PCR) with specific primers for the *speA, speC, speG, speH, speI, speJ, ssa*, and *smez* genes as described previously (*31**–**33*). In addition, a primer pair specific to a DNA region encoding the 23S rRNA (*33*) was used as a positive control.

Recombinant forms of SPE-A, SPE-C, SPE-G, SPE-H, SPE-I, SPE-J, SSA, SMEZ-1, and SMEZ-2 were produced in *Escherichia coli* by using the pGEX-2T expression system as described previously (*31**,**33*). New Zealand white rabbits were immunized with 50 μg of recombinant protein in 1-mL phosphate-buffered saline and 1-mL incomplete Freund’s adjuvants (Invitrogene, San Diego, CA) followed by a booster injection 4 weeks later. The rabbits were bled 10 days after the booster injection. SMEZ-1 and SMEZ-2 were injected as a 1:1 mixture to ensure generation of antibodies against a large panel of the SMEZ variants.

### Western Blot Analysis

*S. pyogenes* isolates were grown overnight in modified BHI medium at 37°C. The medium was prepared by dialyzing 100-mL of 10X concentrated BHI medium against 1 L water. Three grams of glucose, 4 g of Na_2_HPO_4_, and 10 mL of the dialysate were then added to the solution outside the tubing, which was used to grow the bacteria. Bacterial cells were spun down, and the supernatant was transferred into a new tube and spun at high speed (10,000 rpm) in a Beckman JA20 rotor for 20 min to remove remaining cells. The proteins were then precipitated from the clear supernatant after the addition of 0.15 V of 50% tri-chloracetic acid and centrifugation at 10,000 rpm for 20 min. The precipitated proteins were resuspended in PBL to 1/50 of the original volume and mixed with an equal volume of 2x sodium dodecyl sulfate–polyacrylamide gel electrophoresis (SDS-PAGE) sample buffer. The pH was readjusted by blowing ammonia vapor onto the sample until the color changed from yellow back to blue. After boiling for 2 min, 10 μL was loaded onto a 12% SDS-PAGE and run along a protein standard (50 ng, 25 ng, 10 ng, and 2 ng of the appropriate recombinant SAg).

The proteins were blotted onto a Hybond-c extra nitrocellulose membrane (Amersham Life Sciences, Little Chalfont, UK) by using Western transfer buffer (10% methanol, 150 mM glycine, 25 mM tris-HCl pH 8.5) and a Semi-phor semi-dry blotter (Hoefer Scientific Instruments, San Francisco, CA). The membranes were blocked with 5% milk powder (Anchor, Auckland, New Zealand) in TBST (120 mM NaCl, 10 mM Tris-HCl ph8, 0.05% Tween 20) for 30 min at room temperature, before incubation with the appropriate rabbit anti-SAg antiserum (1:5000 in TBST) overnight at 4°C. The blots were developed by using the ECL Western Blotting Analysis System (Amersham Biosciences, Little Chalfont, UK)

## Results

### Mitogenic Activity in Samples from Patients with STSS

Nineteen acute-phase serum samples from patients with severe invasive streptococcal disease with STSS (n=9) and without STSS (n=10) were analyzed for mitogenic activity. Serum samples 96/2 and 99/1 both induced a high proliferative response when tested in a PBL-stimulation assay, which suggests that they might contain a SAg. Both patients had bacteremia and STSS. Peritonitis was diagnosed in patient 96/2, who recovered after 2 weeks in the intensive-care unit; TSS was diagnosed in patient 99/1, who died on the day of admission.

**Figure 1:**
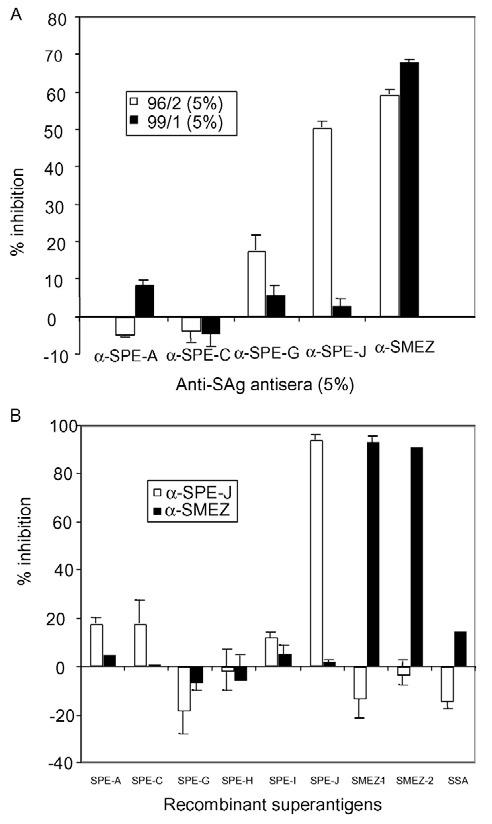
Inhibition of mitogenic activity in sera 96/2 and 99/1 with anti-superantigen (SAg) antisera. A) Peripheral blood lymphocytes (PBLs) were stimulated with 5% patient serum in the presence of 5% anti-SAg antiserum or 5% fetal calf serum (FCS) only. After 3 days, ^3^[H]-thymidine was added, and PBLs were incubated for another 24 h, before being washed and counted. The results were blotted as percentage of inhibition with specific anti-SAg serum compared to FCS. Antistreptococcal pyrogenic exotoxin (SPE)-J antiserum inhibited the mitogenic activity of serum 96/2 by 51%, while antistreptococcal mitogenic exotoxin (SME) Z antiserum inhibited the activity in 96/2 by 59% and the serum in 99/1 by 68%. B) The specificities of the anti–SPE-J and anti–SMEZ sera were demonstrated by stimulating PBLs with recombinant SAgs in the presence of 5% antiserum. SSA, streptococcal superantigen.

To identify the SAg responsible for the mitogenic activity, PBLs were stimulated with serum 96/2 and serum 99/1, respectively, together with rabbit antibodies against recombinant forms of SPE-A, SPE-C, SPE-G, SPE-J, or SMEZ (Figure 1A). Addition of anti-SMEZ antibodies resulted in 59% and 68% inhibition of the mitogenic activity of serum samples 96/2 and 99/1, respectively. Antibodies against SPE-J inhibited PBL stimulation of serum 96/2 by 51% but had no substantial effect on the serum 99/1 induced activity. Anti–SPE-A, anti–SPE-C, and anti–SPE-G antibodies did not substantially inhibit the activity in both serum samples, except for a slight inhibition (18%) of serum 96/2 activity by anti–SPE-G antibodies.

**Figure 2:**
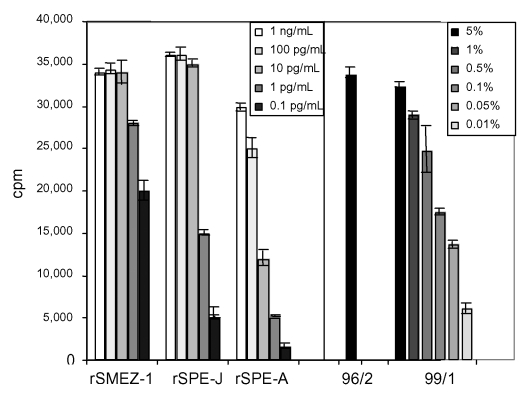
Mitogenic activity of acute-phase serum samples 96/2 and 99/1 compared to recombinant superantigens (SAgs). Peripheral blood lymphocytes were stimulated for 4 d with various dilutions of recombinant SAg or acute-phase serum sample 99/1. No dilution was carried out for 96/2 because of limited amount of serum. Five percent of each of the patient serum samples showed a proliferative response equal to 1–10 pg/mL of recombinant streptococcal pyrogenic exotoxin J or recombinant streptococcal mitogenic exotoxin 1. Serum 99/1 still showed significant mitogenic activity at 0.05%. SME, antistreptococcal mitogenic exotoxin; SPE, antistreptococcal pyrogenic exotoxin.

The antisera were selective against their individual target SAg. Anti–SPE-J and anti-SMEZ antiserum were added to PBLs stimulated with various recombinant SAgs (SPE-A, SPE-C, SPE-G, SPE-H, SPE-I, SPE-J, SMEZ-1, SMEZ-2, and SSA), and results showed that anti–SPE-J antibodies exclusively inhibited the rSPE-J activity, whereas anti-SMEZ antibodies inhibited the activity of rSMEZ-1 and rSMEZ-2 (Figure 1B). To quantify the levels of SAg in 96/2 and 99/1 serum samples, a comparison was made against a standard PBL proliferation response for recombinant SMEZ-1 and rSPE-J. Five percent of each serum resulted in 33,000–34,000 cpm after ^3^[H]-thymidine uptake, which is equivalent to 1–10 pg/mL of rSMEZ-1 or rSPE-J (Figure 2). The 99/1 activity was titratable and still detectable at 0.05% serum. Insufficient 96/2 serum prevented a similar dilution assay.

### STSS Patient 96/2 and Seroconversion to SMEZ

**Figure 3:**
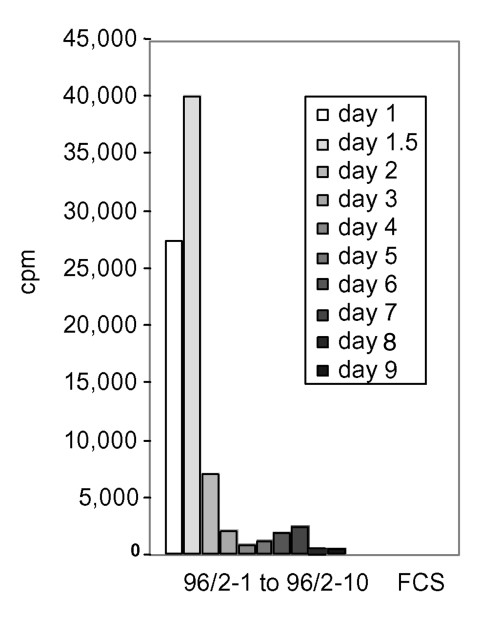
Clearance of the mitogenic activity in sequential sera from patient 96/2. Peripheral blood lymphocytes were stimulated with 5% of acute-phase and sequential serum samples from patient 96/2. The mitogenic activity reached the highest point on day 1.5 after admission to hospital and dropped sharply on day 2. No substantial activity was found in sequential serum samples from day 3 on (samples 96/2–4 to 96/2–10). FCS, fetal calf serum.

Sequential serum samples from STSS patient 96/2 up to day 9 after admission to hospital were analyzed for clearance of mitogenic activity. Figure 3 shows that the highest mitogenic activity occurred 1.5 days postadmission (serum 96/2-2). At day 2 postadmission, an 80% reduction of activity had occurred, and by day 3 and day 9 after admission SAg activity was undetectable. Patient 99/1 died on the day of admission, which prevented sequential serum analysis.

**Figure 4:**
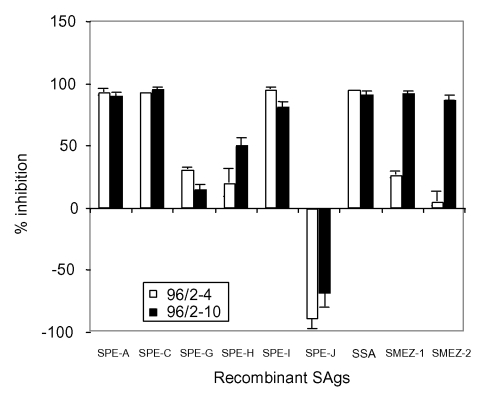
Seroconversion of patient 96/2 against streptococcal superantigens (SAgs). Peripheral blood lymphocytes were stimulated with various recombinant streptococcal SAgs in the presence of serum 96/2–4, 96/2–10, or fetal calf serum only. The columns show the percentage of inhibition of recombinant SAgs by neutralizing antibodies in patient serum samples. The sequential serum on day 3 showed a complete lack of neutralizing antistreptococcal mitogenic exotoxin (SME) Z antibodies, while serum 96/2–10 converted to a high anti-SMEZ antibody titer. Both sera enhanced the mitogenic activity of recombinant streptococcal pyrogenic exotoxin J, which suggests the presence of an unknown synergistic factor. SPE, antistreptococcal pyrogenic exotoxin; SSA, streptococcal superantigen.

The sequential serum samples from patient 96/2 allowed us to analyze for the development of neutralizing anti-SAg antibodies by using patient serum to inhibit the activity of recombinant SAgs in a PBL-stimulation assay (Figure 4). On day 3, patient 96/2 had neutralizing antibodies against SPE-A, SPE-C, SPE-I, and SSA but undetectable levels of protective antibodies against SMEZ-1, SMEZ-2, SPE-G, and SPE-H. By day 9 after admission (sample 96/2-10), the serum contained high titers of neutralizing antibodies against SPE-A, SPE-C, SPE-I, SSA, SMEZ-1, and SMEZ-2, and a moderate anti–SPE-H titer. No antibodies against SPE-G were detected. These results show seroconversion for SMEZ-1 and SMEZ-2 antibodies in the STSS patient 96/2, adding further evidence that SMEZ was the predominant SAg causing STSS in this patient.

Addition of serum 96/2-4 or 96/2-10 selectively increased the mitogenicity of SPE-J by twofold compared to the FCS control. This selective increase suggests that the serum might contain a substance that selectively synergizes SPE-J activity, which prevented any evaluation of the anti–SPE-J antibody titer in the two sequential serum samples.

Serum 96/2-10 (10 days after admission) was analyzed for protective antibodies against SMEZ-1, -2, -3, -4, -5, -7, -8, -9, -13, -16, -20, -21, and -22 and found to neutralize the activity of all tested SMEZ variants (data not shown). Inhibition of approximately 95% was seen with all SMEZ variants, except SMEZ-2 (88%), SMEZ-16 (78%), and SMEZ-22 (85%), indicating that challenge with a single SMEZ variant resulted in a cross-reactive antibody response against all SMEZ variants.

Matching GAS isolates from all 19 patients were genotyped for *sag* genes. The frequencies were 100% (*smez, speG, speJ*), 36.8% (*speA*), 31.6% (*ssa*), 26.3% (*speH*), 15.8% (*spC*), and 10.5% (*speI*) (Table). We observed no difference in *sag* gene frequencies between patients with STTS and patients without STSS. The *smez* alleles of both GAS strains isolated from patients 96/2 and 99/1 were analyzed by DNA sequencing and identified as *smez-1* (H297) and *smez-16* (H360).

All GAS isolates were grown in liquid culture, and the supernatants were analyzed for secreted SAgs by Western blot using rabbit antisera against individual recombinant SAgs (Table). The selectivity of each antiserum was tested with the complete panel of recombinant SAgs in Western blots, and no cross-reactivity was observed (data not shown). SPE-A was expressed in substantial amounts only from isolates from patients with STSS (isolates H325, H330, and H366). Isolates from patients without STSS that carry the *speA* gene (H308, H314, and H315) had undetectable levels of SPE-A. However, patients infected with the SPE-A–producing strains did not show any mitogenic activity in their acute-phase serum samples. In contrast, GAS isolated from the patients 96/2 and 99/1 (H297 and H360) produced only small amounts of SMEZ in vitro despite the relatively large amounts detected in the acute-phase serum samples. In vitro–produced SMEZ could not be detected in Western blots, indicating a concentration of <1 ng/mL, and could only be detected using the more sensitive Jurkat cell proliferation assay that has a lower sensitivity threshold (approximately 10 pg/mL).

## Discussion

Over the last 2 decades, a large increase in GAS-mediated severe invasive disease has occurred (*5**,**24**,**25*). Streptococcal SAgs have been implicated in STSS and other severe streptococcal infections. Evidence for SAg involvement in these diseases derived from studies showing higher frequencies of *speA* and *speC* genes in severe disease isolates compared to nonsevere disease isolates (*2**,**22**–**25*), and reports of strong proliferative responses in the acute-phase serum samples from a patient with STSS (*28*) and from mice infected with a SAg-producing *S. pyogenes* strain (*29*).

In this study, we analyzed 19 acute-phase serum samples from patients with severe streptococcal disease with STSS (n=9) and without STSS (n=10) for mitogenic activity and detected SMEZ in samples from 2 STSS patients (96/2 and 99/1). One of the serum samples (96/2) also contained detectable amounts of SPE-J.

The mitogenic activity in the two serum samples was equivalent to 1–10 pg/mL and 10 pg/mL of SMEZ-1 or SPE-J, which is sufficient to trigger maximal T-cell activation in PBL-stimulation assays. However, this concentration remains an estimate as inhibitory effects of serum components were not defined.

The sequential serum of the surviving patient (96/2) showed no protective anti-SMEZ antibodies on day 3 after admission (serum 96/2–4) but substantial levels at day 9 (serum 96/2–10), which suggests a direct role of this toxin in the STSS of this patient. Challenge with SMEZ-1 (the SMEZ variant produced by GAS isolate H297 from patient 96/2) resulted in a broad neutralization response against the complete range of SMEZ variants. We have shown previously that some variation in healthy blood donors exists in neutralizing antibodies to SMEZ variants because of antigenic variation (*33*). Whether broad-spectrum anti-SMEZ responses are a result of a few cross-reacting antibodies that were raised against a single SMEZ variant has yet to be established, as does whether it results from infection by multiple GAS strains carrying different *smez* alleles over a certain period. Our results indicate the production of cross-reactive SMEZ antibodies in patient 96/2. However, this response might not reflect a general response, and SMEZ concentration in the blood might play a critical role.

Analysis of the 19 matching GAS isolates showed no bias in *sag* genotype between STSS isolates and non-STSS isolates. Furthermore, the results are consistent with the *sag* gene frequencies previously observed in 39 *S. pyogenes* isolates from New Zealand patients with sore throat (n=29), wound infection (n=4), acute glomerulonephritis (n=1), otitis (n=1), endometritis (n=2), rheumatic fever (n=1), and skin ulcer (n=1) (*36*). The only substantial difference was the frequency of the *speC* gene, found in 42% of New Zealand isolates and 15.8% of London isolates. These results suggest that genotyping of *sag* genes alone is not predictive for type or severity of streptococcal disease.

We found no substantial difference in SAg production in cell cultures between STSS and non-STSS strains, with the exception of SPE-A, which was only expressed in STSS strains and suggests a tight association of SPE-A in some cases of STSS. These data are consistent with data from other groups that showed a link between SPE-A expression and severe streptococcal disease (*17**,**22**,**23*). Evidence points to very different regulation of SAg production between in vitro culture and in vivo infection. SMEZ-1, for example, was undetectable in cultures of isolate H297 except by the sensitive Jurkat proliferation assay, yet the patient with this GAS isolate had high serum levels of SMEZ-1, indicating that SMEZ production is tightly regulated. Kazmi et al. showed the in vivo induction of the *speA* gene by using a micropore Teflon diffusion chamber implanted subcutaneously in BALB/c mice (*37*). Broudy et al. observed an induction of SPE-C after they co-cultured a *speC*-carrying isolate with a human pharyngeal cell line (*35*). Some, if not all, SAgs can likely be induced or upregulated by an unknown host factor.

Despite the fact that SMEZ is poorly expressed in vitro while most other SAgs are frequently produced in culture, SMEZ appears to be the predominant bioactive circulating SAg at the time of admission. This observation is in keeping with recent findings using isogenic *smez+* and *smez-* bacteria in a mouse model (*29*).

We attempted to correlate streptococcal disease and disease severity with the production of a particular SAg. Although the patient cohort is small, no direct correlation between disease severity and *sag* genotype/SAg production is evident; other factors likely contribute to STSS severity. Importantly, host factors may enhance the production of particular SAgs and, in addition, the immunogenic background of the host may contribute to the severity of SAg-mediated invasive streptococcal disease, as recently reported (*38*). Although the overall potential role of SAgs in severe streptococcal disease remains elusive, our results directly implicate SMEZ in the onset of STSS in at least two patients. This presence of a particular bioreactive SAg in the blood of patients with a reemerging potentially fatal disease is new and provides a platform for further investigation of the role of this potent SAg in disease pathophysiology.
